# Anti-fertility effect of *Aerva lanata* crude extract in male Dams offspring: An experimental study

**DOI:** 10.18502/ijrm.v21i3.13199

**Published:** 2023-04-14

**Authors:** Raphael Eguono Uwejigho, Kingsley Afoke Iteire, Felix Udawmojo Enemali

**Affiliations:** Department of Anatomy, Faculty of Basic Medical Sciences, University of Medical Sciences, Ondo State, Laje Campus, Ondo City, Ondo State, Nigeria.

**Keywords:** Amaranthaceae, Fertility, Male fertility, Male reproductive system, Local herbs.

## Abstract

**Background:**

*Aerva lanata, *a herb used as food and also consumed as a tonic by pregnant women to relieve stomach pains and prevent miscarriage. In addition to other characterized properties, it possesses antifertility and anti-implantation activities.

**Objective:**

This study investigates the testicular toxicity of the testes of offsprings of Dams treated with crude aqueous extract of *Aerva lanata*.

**Materials and Methods:**

25 pregnant Wistar rats (Dams) weighing 180-240 gr were randomly earmarked into 5 groups (n = 5/each). Group A served as control; groups B, C, D, and E received 200, 400, 800, and 1000 mg/kg body weight of *Aerva lanata* extract*,* respectively, beginning from 12 ${}^{\text{th}}$ to 19 ${}^{\text{th}}$ day of gestation. The pups (delivered of Dams) were weighed, observed, and sacrificed 6 wk post-parturition. The testes of the male pups were obtained for histological procedures the testis histology was examined.

**Results:**

No gross malformation was observed in the treatment groups, the number of pups/litter was significantly reduced in group E (p= 0.01), pups weight analysis showed a significant reduction in groups C and E (p= 0.04, and 0.02 respectively), and the mean pup testes weight was significantly reduced in groups B, C, D, and E (p= 0.03, 0.03, 0.01, and < 0.001 respectively) when compared with control. Histologically, the treated pup testes tissues showed varying degrees of disruption and distortion of the cellular arrangements of the germinal epithelium in a dose dependent manner compared to the control.

**Conclusion:**

The study revealed a testicular toxicity and possibly antifertility role of *Aerva lanata* in dams' pups.

## 1. Introduction

Medicinal plants have a long history of use in traditional medicine worldwide (1), being the basis for treating many ailments and diseases in African traditional medicine practice. This is due to several reasons, including availability, affordability, accessibility, and promising efficacy compared to the often-high cost and adverse effects associated with standard synthetic drug agents (2, 3). In the traditional health system of most communities, most of these medicinal plants are used without empirical knowledge of their side effect on the human system. Usage was based on the knowledge passed down from oral history and traditions of forebears who might not have observed the side effects of the drugs on other organs in the human body (3).

*Aerva lanata *is a woody perennial herb of the genus *Aerva* in the Amaranthaceae family. In Nigeria, the Yorubas refer it to as Ewi-owo or Ewe Aje, while the Hausas call it Furfurata. Ithas been reported to be used to treat kidney-related infections (4), fever, guinea worm infection, diabetes, bleeding from a cut, and eye disease (5). Extracts made of *Aerva lanata* are given to pregnant women as a tonic to relieve stomach-pains and prevent miscarriage (4-6). The aerial portion of *Aerva lanata* were extracted with ethanol, and appraised for its antifertility attribute employing anti-implantation, aborticide, and spermatozoa motility of rat (in vitro) models. The anti-implantation upshot appears to be dose-dependent, as well as the time of treatment initiation during pregnancy; pre-implantation loss was observed. At a concentration of 10%, *Aerva lanata* exhibited no motility of spermatozoa (rats) within 60 sec (7).

Literatures on the possible teratogenic effect of this extract are scarce. At definite critical periods of pregnancy, embryos are hypersensitive to agents responsible for aberrant development than at other times. The period of ultimate vulnerability to aberrant development (in humans) transpire between wk 3 and 8, this is when stem cells begin to differentiate into functional cells (8). With cases of congenital abnormalities attributed to traditional herbal preparation consumed during pregnancy (9), it became imperative that this research is carried out to determine the possible teratogenic effect of *Aerva lanata *on the testes of offsprings of treated Dams.

This study canvasses the effects of *Aerva lanata* on the testis of offsprings of treated Dams. To actualize this, the investigation was done on the upshots of the aqueous extract of *Aerva lanata *on Dams administered on specific periods of pregnancy, on litter size, body weight, and testes weight of male pups, and the histology of the testes of 6 wk old offsprings of treated Dams. This study will contribute to the safety of consuming traditional herbal remedies during pregnancy.

## 2. Materials and Methods

### Plant collection and identification

In this experimental study, the leaves of the *Aerva lanata* plant were purchased from victory line 28 at New Benin market, Benin City, Nigeria. The plant was identified in the Department of Pharmacognosy, Faculty of Pharmacy, University of Benin, Benin City, Nigeria. A voucher specimen was placed in the Herbarium of the Department.

#### Extract preparation

*Aerva lanata *leaves were air-dried at 27C for 8 wk, and then pounded with a wooden mortar and pestle and processed into fine powder. Extracted was done on the powdered specimen using distilled water by percolation for 24 hr. The concoction was filtered, and employing a vacuum rotary evaporator, evaporation was done on the filtrate at 40C. The residue was stored in a refrigerator at the Department of Anatomy, University of Benin, Benin City, Nigeria. On each experiment day, a fraction of the crude extract was dissolved in distilled water for use (10-12).

### Care and management of animals

25 adult female Wistar rats weighing 180-240 gr were purchased from the animal facility of the Department of Anatomy, School of Basic Medical Sciences, University of Benin, Benin City, Nigeria. The animals were kept in the animal house of the University of Benin, Benin City, Nigeria. The animals were allowed to acclimatize for 2 wk before the commencement of the experiment. During acclimatization, animals were allowed access to Growers' mash (produced by Livestock Feed Company, Benin City) and water *ad libitum.*

### Mating of animals and experimental design

The estrous cycle of female rats was monitored daily by viewing their vaginal smears under the microscope. Female rats in their proestrous phase were paired in a cage with male rats overnight in a ratio of 1:1. At the break of dawn, smears were taken from the vagina of the female rats and observed under the microscope for the presence of sperm cells or copulatory plugs. Smears from animals with sperm cells were assumed to be pregnant and considered as embryonic day 1 (E1). The Dams (Pregnant Wistar Rats) were distributed into 5 (5) groups. Each group is comprised of 5 Dams. Dams were randomly assigned into a control group (A) and 4 treatment groups (B, C, D, and E). Dams in the treatment groups were administered with different doses of aqueous extract of *Aerva lanata *on days 12 to 19 of pregnancy, which is the critical period for testes development in rats. Animals in groups B, C, D, and E received 200 mg/kg, 400 mg/kg, 800 mg/kg, and 1000 mg/kg body weight of aqueous leaf extracts of *Aerva lanata,* respectively. On the day of birth, both the pups and mothers were examined for gross malformation, the number of pups per litter, and their weight (13, 14).

### Sacrificing animals and dissection of organs

By the 6 ${}^{\text{th}}$ wk of life, the pups were sacrificed using cervical dislocation. A midline incision was made via the ventral wall of the abdomen, the testes were dissected out, weighed, and fixed in Bouin's fluid for routine histological examination.

### Tissue processing 

Standard histological techniques were employedfor the testis tissue processing and staining (15, 16) as described below.

The testes tissue were cut down to about 3-5 mm thick sections and treated using the paraffin wax embedding method as described: water was removed from sections for 1 hr each at 27C employing ethanol in ascending grades: 70% ethanol, 90% ethanol, Absolute ethanol I and absolute ethanol II. The dehydrated tissues were cleared in 2 changes (1 hr each) of xylene, followed by infiltration in 2 changes (1 hr each) of molten paraffin wax at 60C and ultimately embedded in paraffin wax multi-block plastic embedding molds. The tissues blocked in paraffin were then cut down and fastened on wooden chocks on a rotary microtome and sectioned.

A rotary microtome (Bright B5143, Huntington, England) was used to cut 5 µm thick sections from the tissues blocks. They were placed in a water bath (40C) to allow the folded ribbons of sections to spread. The sections were secured on clean glass slides and dried at 40C on a slide drier.

#### Hematoxyline and eosin staining procedures

The sections were dewaxed in 2 changes of xylene for 2 min each and rehydrated in descending grades of alcohol (absolute I, absolute II, 95%, 90%, 70%, and 50% ethanol) for 2 min each. The sections were then rinsed in distilled water for 3 min and stained in hematoxylin for 20 min. Excess hematoxylin stain was removed by rinsing well under running tap water for 2-3 min and differentiated in acid alcohol (0.5% HCL in 70% ethanol) for 3 sec. The sections were further rinsed well under running water for 15 min to remove acid alcohol, counterstained in 1% aqueous eosin for 2-4 min, dehydrated rapidly in ascending grades of ethanol (50% through absolute ethanol), cleared in xylene and mounted in a synthetic resin medium using clean glass cover slip.

### Photomicrography

The tissue sections were canvassed with a microscope (Leica DM750) with an attached Leica ICC50 digital camera. Photomicrographs of the tissue sections were considered at diverse magnifications.

### Ethical considerations

Before the commencement of this experimental research, Ethical approval was obtained from the University of Benin Research Ethics Committee, Office of Research Management and Development Benin City, Nigeria. (Code: UNIBEN/REC/MM55/01231). Care of animals was done following the Guidelines for the Care and Use of Laboratory Animals prepared by the National Academy of Sciences for the user of laboratory animals (17).

### Statistical analysis

Descriptive and inferential statistics were used to canvass the data garnered. The values were exhibited as mean ± Standard Error of means. Statistical Package for Social Sciences (version 17.0 for windows (SPSS Inc, Chicago, II, United States) was employed for all statistical analysis. One-way analysis of variance (ANOVA; 95% confidence interval) was employed to ascertain the significance of the difference in the means of all parameters for more than 2 sets of data comparison and for 2 sets of data, *t *test was employed. Dunnett multiple comparisons for all groups and with control was employed for post hoc test. A p-value < 0.05 was considered statistically significant.

## 3. Results 

### Effects of *Aerva lanata* on pup testes weight, pup weight, and litter size

**Figure 1:**
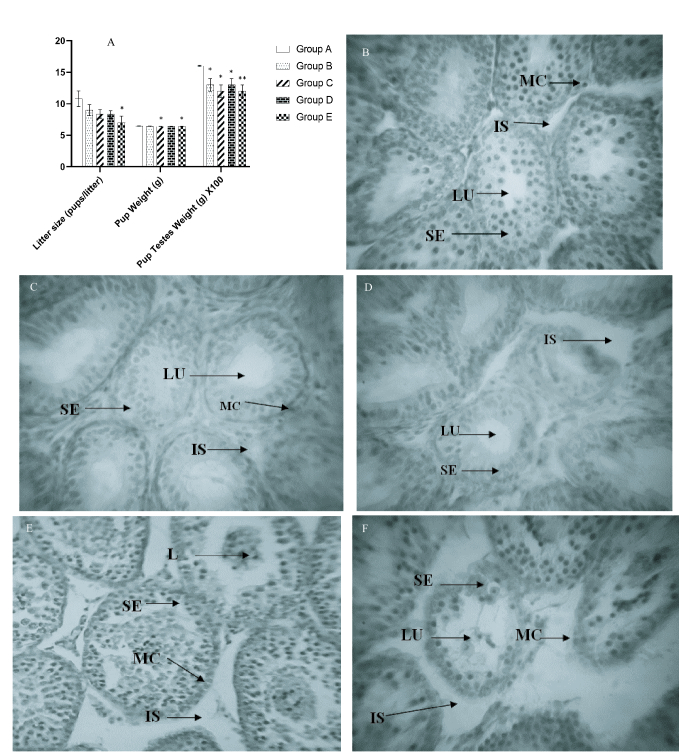
A) Effects of crude aqueous extract of *Aerva lanata* on litter size, pup weight and pup testes weight. The Mean ± SEM are represented in the Chart, n = 5, *indicates significant difference at p < 0.05, **p < 0.01 compared with control. B) Photomicrograph of control showing juvenile seminiferous tubules cut in various planes of sections and the interstitial tissue of the testis. Each seminiferous tubule is lined by connective tissue containing myoid cells (MC), seminiferous epithelium with spermatogenic (SE), lumen of the seminiferous tubule (LU), and interstitial space (IS). C) Photomicrograph of group B, each seminiferous tubule is lined by connective tissue containing myoid cells (MC), seminiferous epithelium with spermatogenic cells in various stages of differentiation (SE), lumen of the seminiferous tubule (LU). D) Photomicrograph of group C shows juvenile seminiferous tubules (SE), interstitial space (IS), lumen of the seminiferous tubule (LU). E) Photomicrograph of group D shows juvenile seminiferous tubules (SE), myoid cells (MC), and interstitial space (IS), lumen of the seminiferous tubule (L). F) Photomicrograph of group E shows juvenile seminiferous tubules and the interstitial tissue of the testis (SE), lumen of seminiferous epithelium (L), interstitial space (IS) (H&E x400).

Figure 1A shows the comparison of pup testes weight, pup weight, and litter size (pups/litter) with different dosages of crude aqueous extract of *Aerva lanata. *The number of pups/litters were not significantly different in groups B, C and D (p = 0.35, 0.15, and 0.15 respectively), but was significantly reduced in group E (p= 0.01), when compared with the control.Pup weight was not significantly different in groups B and D (p= 0.21 and 0.69 respectively) but was significantly reduced in groups C and E (p = 0.04 and 0.02 respectively) when compared with control. *Aerva lanata* significantly reduced testes weight in groups B, C, D, and E (p= 0.03, 0.03, 0.01, and < 0.001 respectively) when compared with control.

### Histologic effects of *Aerva lanata* on the testes 

The testicular tissue sections of control group (A) rats showed seminiferous tubules with numerous strata of spermatogenic cell series, the lumen of the seminiferous tubule, myeloid cells, and the interstitial space. These features appear normal, as shown in figure 1B below. The testes of Dam offsprings in group B (treated with 200 mg/kg body weight of aqueous extract of *Aerva lanata*) showed no observable histological changes compared with control, as shown in figure 1C. Dams in group C (treated with 400 mg/kg body weight of crude aqueous extract of *Aerva lanata*) showed mild degeneration of spermatogonia in the seminiferous epithelium, mild interstitial edema, and loss of sequential arrangement of the seminiferous epithelium (Figure 1D). Testes of Dams in group D (treated with 800 mg/kg body weight of crude aqueous extract of *Aerva lanata*) (Figure 1E) showed cytoarchitectural distortion and disruption of the arrangement of spermatogonia in the seminiferous epithelium, with gametogenic cells occupying the lumen of the seminiferous tubule and widening interstitial space. Group E (treated with 1000 mg/kg body weight of crude aqueous extract of *Aerva lanata*) showed disruption of gametogenic cells in the seminiferous epithelium, with cellular debris in the lumen of seminiferous tubule and widening interstitial space (Figure 1F).

## 4. Discussion

In this study, a significant decrease was observed in the litter size (pups/litter) when Dams were administered with 1000 mg/kg body weight of *Aerva lanata.* This decrease may have been due to its reported abortifacient effect (16), causing the abortion of fetuses. We also observed a statistically significant decrease in body weight of offspring from Dams treated with 400 mg/kg and 1000 mg/kg body weight, while the pup testes weight was significantly decreased in all groups that received *Aerva lanata* extracts. Although information on the effects of *Aerva lanata* on juvenile testes is limited, there is data indicating that the crude extracts of *Aerva lanata* possess anti-fertility, anti-implantation, and abortifacient effects at a dose range of 200-400 mg/kg (16, 18). A study that investigated the toxicity profile of the aqueous extract of *Aerva lanata*, demonstrated that at 1000 mg/kg, the extract caused a significant reduction in sperm count and motility of male rats (19).

Histopathologic examination of juvenile testis is necessary to determine the safety of new drugs and the end point in male pubertal development (14). In this study, examination of the histological architecture of the juvenile testes (Figure 1B) shows the normal architecture of a juvenile testis in the control group; this is characterized by seminiferous tubules cut in various planes of sections and the interstitial tissue of the testis. Each seminiferous tubule is lined by connective tissue containing myoid cells. The seminiferous epithelium is visible with spermatogenic cells in various stages of differentiation; the seminiferous tubule's lumen appears empty and devoid of tufty tails of spermatozoa, indicating its juvenile nature. The spermatogenic cells seen in this plate consist mainly of spermatogonia and earthly stages of the spermatogenic series at different stages of differentiation.

The administration of 200 mg/kg body weight of crude aqueous extract of *Aerva lanata *to the Dams (Figure 1C) also showed a normal architecture of the juvenile testis, characterized by seminiferous tubules cut in various planes of sections and the interstitial tissue of the testis. The seminiferous tubules are lined by connective tissue with myoid cells. Also, the seminiferous epithelium shows spermatogenic cells in various stages of differentiation. The lumen of the seminiferous tubule is empty and devoid of tufty tails of spermatozoa. *Aerva lanata* have been documented to be an asset bestowed by nature due to its numerous remedial qualities. However, there are drawbacks to these all-encompassing beneficial qualities. The implantation loss, abortive properties and, sperm motility was assessed on the roots and aerial parts of the plant, using doses of 200 and 400 mg/kg body weight. The study documented a 20% and 30% pre-implantation loss and a pregnancy failure of 30% and 40%, respectively. This study also showed no movement of rat spermatozoa within 60 se of administration of 10% concentration of the extract (20). This anti-fertility attribute of was also observed when we administered 400 mg/kg body weight of the crude aqueous extract, mild degeneration of the spermatogenic series in the seminiferous epithelium, mild interstitial edema, and loss of sequential arrangement of the seminiferous epithelium were observed as indicated (Figure 1D).

Disruption of early spermatogenic series in the seminiferous epithelium, with spermatogenic cells occupying the seminiferous tubule's lumen, was observed when 800 mg/kg body weight of crude aqueous extract of *Aerva lanata *was administered to the Dams (Figure 1E). There was also the widening of the interstitial spaces at this dosage. The mechanism of action exhibited by medicinal plants with antifertility attribute varies, notably is their suppressing influence on fertility by direct action the on sex hormones (21). Those exhibiting estrogenic attribute influence the pituitary action by reducing the secretions of luteinizing hormone and follicle stimulating hormone, thereby disrupting fertility. In females, the hypothalamus, anterior pituitary, and ovary are the action site for anti-fertility agents, with the uterus being the major target (22). In males that are past puberty, the function of the testes is gravely determined by the activities of the gonadotropins. Testosterone is produced by Leydig cells because of the stimulation of luteinizing hormone, together with follicle stimulating hormone, testosterone indirectly influence spermatogenesis by regulating Sertoli cell function and inhibiting secretion (23). However, in the fetus, the role of pituitary-derived hormones is not very clear. Besides, placental gonadotropin has been observed to be important for normal fetal testicular development, mostly in primates (24).

Typical estrogenic compounds can also cause ova expulsion from the tube, disrupt blastocyte luteotropic process and equilibrium function between endogenous estrogen and progesterone, thus distorted fertility as an upshot (22). Anti-implantation outcome is induced in immature rats with estrogenic compounds through increasing the uterine wet weight and inducing cornification and opening of the vagina (21). In males, the antifertility action of medicinal plants is attributed to their antispermatogenic or antisterioidogenic properties of one or more active ingredients (25). In this case, the anti-implantation properties of *Aerva lanata* are attributed to its alkaloids, kaempferol, quercetin, B-sitosteryl acetate and tannic acid constituents (26). Noteworthy in our dose of 1000 mg/kg body weight (Figure 1F), it resulted in marked disruption of seminiferous epithelium, cellular debris in the lumen of the seminiferous epithelium, tissue edema and widening of the interstitial spaces between the seminiferous tubules. The role of *Aerva lanata* on male fertility at this dose considered in our research is similar to result obtained from a previous study where the extract significantly reduced sperm count and motility of male animals. At this dosage, mild to moderate cellular changes in the testes have also been reported on treated rats when ascertained on histological examinations (19) disclosing its toxic attributes of cellular damage to organs and diminution in male reproductive capability.

However, to the best of our knowledge at the time of this research, no other work was available on the teratogenic effect on *Aerva lanata,* as such the comparison of the histological architecture of *Aerva lanata* on juvenile testes with other research was limited.

### Study limitations

The scope of this study is limited to morphological and histological examinations of the testes of offspring of Dams treated with *Aerva lanata*.

## 5. Conclusion

The present study showed that the administration of the crude extract of *Aerva lanata *at different doses at the critical period of organogenesis during gestation disrupted the architecture of the spermatogenic cell series of the seminiferous epithelium of the juvenile testes of Wistar rats. There was also tissue edema and widening of the interstitial spaces between the seminiferous tubules, as indicated. These findings may have implications for consumption of *Aerva lanata* during pregnancy. The implications of these findings for human fertility and gestation require further investigation.

##  Conflict of Interest

The authors declare that there is no conflict of interest.
